# VEGF_111_: new insights in tissue invasion

**DOI:** 10.3389/fphys.2015.00002

**Published:** 2015-01-22

**Authors:** Kevin Danastas, Valery Combes, Laura A. Lindsay, Georges E. R. Grau, Michael B. Thompson, Christopher R. Murphy

**Affiliations:** ^1^Discipline of Anatomy and Histology, School of Medical Sciences, Bosch Institute, The University of SydneySydney, NSW, Australia; ^2^Discipline of Pathology, School of Medical Sciences, Bosch Institute, The University of SydneySydney, NSW, Australia; ^3^School of Biological Sciences, The University of SydneySydney, NSW, Australia

**Keywords:** vascular endothelial growth factor, angiogenesis, cancer, metastasis, tumorigenesis, placentation

## Abstract

Vascular endothelial growth factor is a secreted glycoprotein that acts on endothelial cells to induce developmental and physiological angiogenesis. It has also been implicated in angiogenesis occurring in several pathologies, most notably, cancer. Alternative splicing of VEGF mRNA transcripts results in several isoforms with distinct properties depending on their exon composition. Recently, a new isoform has been identified, VEGF_111_ with a unique exon composition responsible for its high angiogenic potential. In humans, the only known inducer of VEGF_111_ is DNA damage but its natural presence in the uterus of the viviparous lizard, *Saiphos equalis*, suggests other mechanisms of regulation. Most interestingly, the possible relationship between the evolution of viviparity and the associated increased risk in developing cancer may be important in understanding the mechanisms underlying tumor development.

## Introduction

Angiogenesis is a crucial process during tumor growth, invasion and metastasis for the rapid development and maintenance of a blood supply to developing tumors (Weidner et al., 1991). Capillaries do not usually actively proliferate under normal conditions in adults, but tumors secrete several growth factors to stimulate surrounding endothelial cells (ECs) to invade, rapidly proliferate and develop a dedicated blood supply (Carmeliet and Jain, 2000).

One of the most important growth factors involved in angiogenesis is vascular endothelial growth factor (VEGF), a major mitogen that acts selectively on ECs to stimulate angiogenesis (Ferrara and Henzel, 1989; Keck et al., 1989). Also referred to as VEGF-A, vascular permeability factor and vasculotropin, VEGF belongs to a family of proteins along with VEGF-B, C, D and placenta growth factor (Ferrara, 2004).

VEGF is widely expressed in human fetal and adult organs, primarily in lung, kidney and spleen, and at lower concentrations in several other major organs. It is vital for both maintenance of the vasculature and stimulation of angiogenesis (Shifren et al., 1994). Cancers have developed strategies to utilize the angiogenic role of VEGF to grow and metastasize, but this rapid and uncontrolled angiogenesis results in the blood vessels within tumors often having abnormal characteristics including tortuosity, random branching and variable lumen size (Gimbrone et al., 1972; Langenkamp and Molema, 2009). A newly discovered splice variant of VEGF, known as VEGF_111_, is produced by human cells with DNA damage and potentially supports the development and metastasis of tumors due to its high angiogenic activity (Mineur et al., 2007) and may provide new insights into how cancers develop.

## Background to a new isoform: biological effects of VEGF

**Figure 1:**
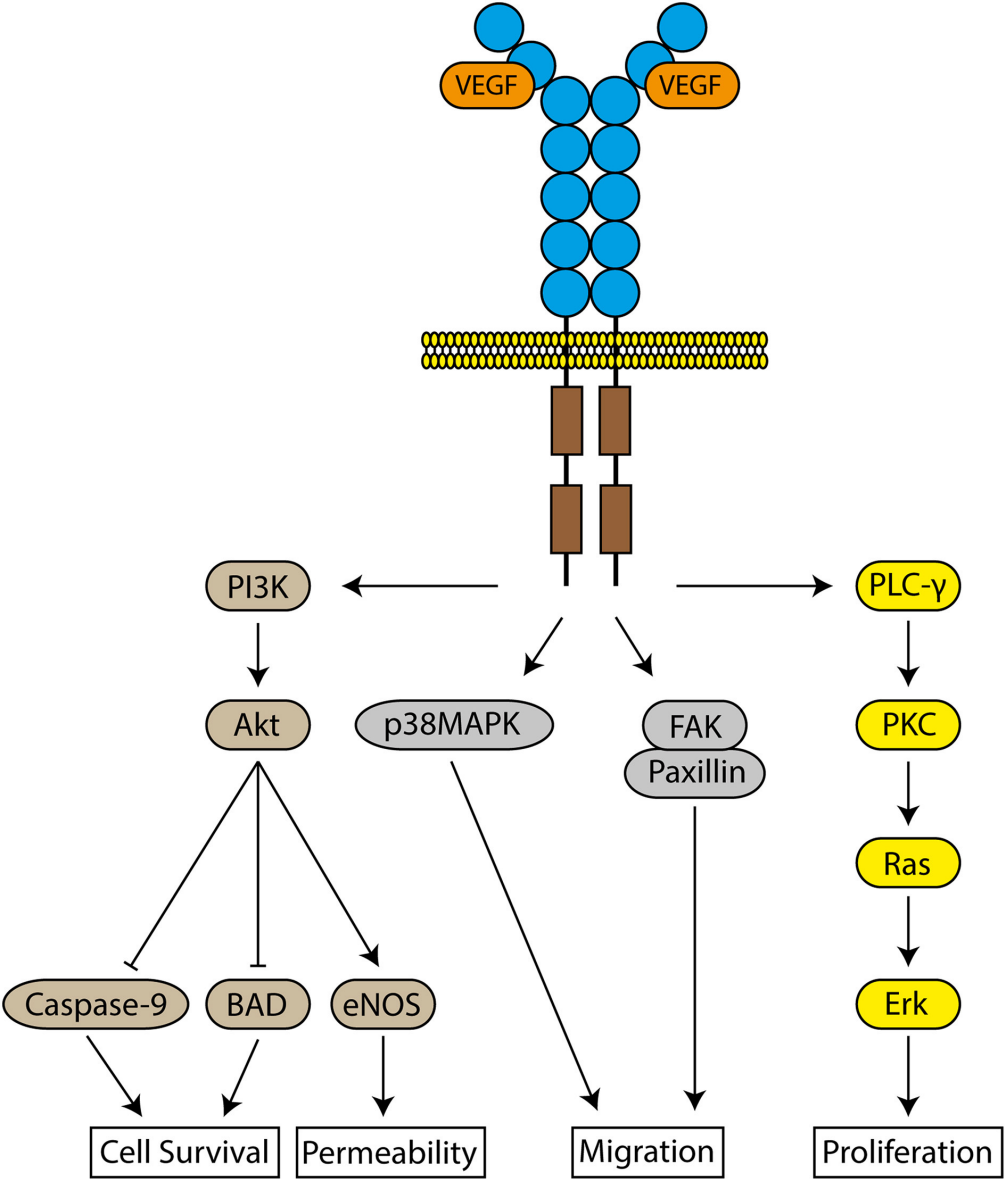
**Signaling pathways of VEGFR-2**. The binding of VEGF to VEGFR-2 results in the activation of several intracellular molecules to induce cell survival, increase vascular permeability, cellular migration and proliferation.

VEGF induces angiogenesis by selectively binding to two tyrosine kinase receptors, VEGFR-1 and VEGFR-2, as well as the neuropilin co-receptor, expressed on the surface of ECs (De-Vries et al., 1992; Terman et al., 1992). Both receptors are essential for normal vascular development, but VEGFR-2 is the major regulator of the biological effects of VEGF via downstream pathways (Shalaby et al., 1995; Olsson et al., 2006) (Figure 1). ECs within tumors are often hypoxic, which induces the overexpression of both VEGFR-1 and 2 on these ECs, contributing to uncontrolled angiogenesis (Veikkola et al., 2000).

EC activation by VEGF induces the production of several proteins, including interstitial collagenase, and urokinase-type and tissue-type plasminogen activators, to facilitate EC invasion into the underlying basement membrane and extracellular matrix (Pepper et al., 1991; Unemori et al., 1992). There is also an increase in vascular permeability (Senger et al., 1983), leading to the extravasation of proteins into the surrounding tissue due to opening intercellular junctions and production of fenestrations between ECs. Tumors exhibit uncontrolled vascular permeability due to the increased incidence of these fenestrations (Roberts and Palade, 1995).

## VEGF gene and isoforms

**Figure 2:**
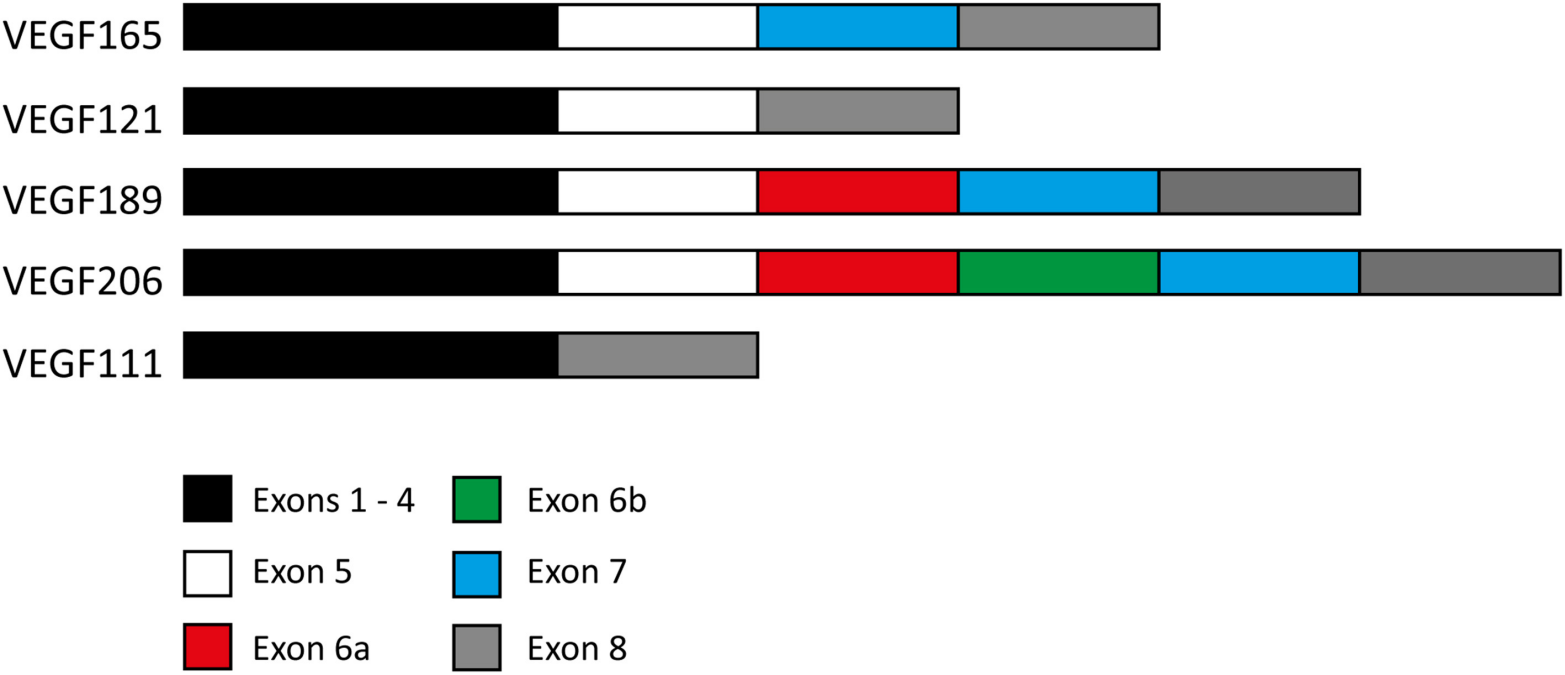
**Exon composition of various VEGF isoforms**. Exon composition of the four naturally expressed isoforms of VEGF in humans. Exons 6 and 7 encode for heparin and neuropilin binding sites. Note the 24 amino acid insertion within VEGF_189_ making up exon 6a, and also the same 24 amino acid insertion followed by a 17 amino acid insertion (exon 6b) in VEGF_206_. The exon composition of the VEGF_111_ isoform is also included for comparison.

The gene for VEGF encodes 8 exons and 7 introns. Exons 1 and 2 encode for the signal peptide and exons 3 and 4 encode for the binding sites to VEGFR-1 and 2 respectively (Robinson and Stringer, 2001). The other exons may or may not be present, resulting in various isoforms. Exon splicing of the mRNA transcript results in four naturally expressed isoforms named after the number of amino acids encoded after signal sequence cleavage (Ferrara, 2004) (Figure 2).

The residues encoded by exons 6 and 7 contain heparin and neuropilin-binding sites, limiting the ability of VEGF_189_ and VEGF_206_, and to a lesser degree VEGF_165_, to diffuse freely upon secretion, resulting in a high proportion remaining bound to the cell surface or surrounding extracellular matrix (Leung et al., 1989). In contrast, VEGF_121_ lacks these binding domains making it freely diffusible (Houck et al., 1992). Whereas VEGF_165_, VEGF_121_, and VEGF_189_ are widely expressed in a range of normal and pathological tissues, VEGF_206_ is rare and has only been identified in the cDNA library of human fetal liver (Houck et al., 1991). Other isoforms have been identified in transformed cells but most recently, the discovery of the VEGF_111_ isoform has raised questions about its involvement in tumor development and metastasis (Mineur et al., 2007).

## The new isoform: VEGF_111_ discovery and identification

VEGF_111_ is encoded by exons 1–4 and 8 (Figure 2) and is induced by DNA damage to human cells caused by ultraviolet B (UV-B) radiation and genotoxic drugs (Mineur et al., 2007) as well as mild hypothermia (Neutelings et al., 2013). The total VEGF mRNA levels are not increased, but rather the proportions of the other VEGF isoforms are altered to accommodate VEGF_111_ production. This is primarily at the expense of VEGF_165_ (Mineur et al., 2007). The concentration of VEGF_111_ also increases with increasing UV-B intensity, presumably because there is a higher rate of genetic damage occurring, in this case the formation of pyrimidine dimers. Genotoxic agents that result in genetic damage through double strand breaks, including captothecin, mimosin and mitomycin C, also induce VEGF_111_ formation.

The only documented evidence of the natural expression of VEGF_111_ is in the uterine wall, testes and kidneys of *Saiphos equalis*, a viviparous lizard from eastern Australia. VEGF_111_ is absent from any other somatic organ tested (Murphy et al., 2010). Uterine expression of VEGF_111_ increases during pregnancy in *S. equalis*. Other isoforms involved in uterine angiogenesis are also expressed within the uterus. Important changes occur in uterine microvascular architecture in *S. equalis* to support the developing embryo, with increased vessel density evident particularly in late stage pregnancy (Parker et al., 2010). No VEGF_111_ mRNA transcripts have yet been identified in any other species (Murphy et al., 2010).

The role of VEGF_111_ in the uterus of *S. equalis* is currently unknown, but may be involved in the rapid angiogenesis of both the uterus and chorioallantoic membrane of the embryo (Murphy et al., 2010). The significance of the natural production of VEGF_111_ in *S. equalis* suggests there are means of regulating its production, other than genetic damage. VEGF_111_ may be under hormonal regulation, possibly by estrogen or progesterone, but the exact nature of VEGF_111_ regulation is unknown.

## Properties of VEGF_111_

VEGF_111_ is angiogenic and will induce vascularization *in vivo* and *in vitro* (Mineur et al., 2007) resulting in the formation of a functional vasculature (Delcombel et al., 2013). VEGF_111_ is unique in that it is resistant to proteolytic cleavage and retains its complete biological activity upon exposure to plasmin, due to not encoding for exon 5, which contains the residues Arg110-Ala111, the site of plasmin cleavage (Keyt et al., 1996). All other isoforms encode for this cleavage site and their biological activity is decreased upon exposure to plasmin (Houck et al., 1992). Like VEGF_121_, VEGF_111_ lacks extracellular matrix binding regions and thus is also freely diffusible (Mineur et al., 2007), which is evident from the widespread vascular permeability induced by VEGF_111_ in comparison to VEGF_165_ (Delcombel et al., 2013). These unique characteristics result in its high angiogenic activity compared to other VEGF isoforms as shown by the ability of VEGF_111_ to promote early blood vessel recruitment and reduce the effects of ischemia and hypoxia in newly grafted tissue (Labied et al., 2013) and improve healing in tendon injuries (Kaux et al., 2014) compared to other isoforms. The high angiogenic activity, and the fact that it is induced by genetic damage, allows VEGF_111_ to support the development of a blood supply to tumors (Mineur et al., 2007; Delcombel et al., 2013).

VEGF_111_ is fully glycosylated, unlike VEGF_165_ and VEGF_121_, which are only partially glycosylated. Glycosylation is important in efficient protein secretion, which occurs with VEGF_111_, but does not affect its biological activity (Mineur et al., 2007). VEGF_111_ also stimulates the migration of ECs but not monocytes. As monocyte chemotaxis is primarily mediated via VEGFR-1, the chemotactic signals stimulated by VEGF_111_ are primarily mediated via VEGFR-2 (Mineur et al., 2007). VEGF_111_ binds to VEGFR-2 with similar affinity to the other isoforms and along with VEGF_121_, is the strongest inducer of VEGFR-2 phosphorylation in the absence of the neuropilin co-receptor, which is needed for complete activation by VEGF_165_ (Delcombel et al., 2013). VEGF_111_ is unable to bind this neuropilin co-receptor due to lacking the important binding domains, but the lack of binding has no impact on its biological activity (Delcombel et al., 2013).

While hypoxia and hypoglycemia induce VEGF expression by increasing the stability of the mRNA transcript (Stein et al., 1995), mammalian cells do not produce VEGF_111_ in these conditions. Apoptosis and reactive oxygen species also do not induce VEGF_111_ expression (Mineur et al., 2007). While inducers of VEGF_111_ expression remain unclear, caffeine, epigallocatechin gallate (an antioxidant extracted from tea) and resveratrol (a phenol produced by some plants) inhibit VEGF_111_ production (Munaut et al., 2010). Thus, the only known inducer of VEGF111 in human cells is genetic damage (Mineur et al., 2007; Neutelings et al., 2013).

## Role of VEGF in cancer

Angiogenesis is critical in the growth of solid tumors, as a tumor cannot grow beyond a size of 2 mm without its own vascular support (Gimbrone et al., 1972). VEGF mRNA is expressed in several types of tumors and inhibiting VEGF inhibits tumor growth *in vivo*, indicating its importance in tumor angiogenesis. Several anti-VEGF therapies have been developed as cancer treatments, some showing success in improving survival rates (review by Jain et al., 2006).

An issue that has recently emerged is the detrimental effects of VEGF inhibitors on treatment progression, particularly regarding increased malignancy and metastasis (Paez-Ribes et al., 2009). The normalization of the vessels, which involves blocking VEGF signaling to repair vascular abnormalities, may be a better treatment option than eliminating the tumor vasculature, reducing metastasis and improving chemotherapeutic treatments (Mazzone et al., 2009).

Genetic mutations, such as those caused by UV-B radiation and genotoxic drugs, are a major cause of cancer, resulting in abnormal cellular functions (Brash et al., 1991). Thus, it is possible that cancers induced by genotoxic actions may produce VEGF_111_. Genotoxic drugs such as campothecin and mitomycin C, both of which induce VEGF_111_ production, are common chemotherapeutics, and thus it is possible that the production of VEGF_111_ during cancer therapy increases drug resistance (Mineur et al., 2007). Therefore, examining the role of VEGF_111_ in angiogenesis and EC activation may provide a significant contribution to understanding this process.

When nude mice are injected with cells expressing equal concentrations of different VEGF isoforms, the vascular structures differ. Tumors expressing VEGF_111_ are poorly vascularized in comparison to tumors expressing other isoforms. However, the vessel density in the surrounding tissue is highest in the VEGF_111_ tumors compared to all other isoforms tested, which have significantly lower vessel density (Mineur et al., 2007; Delcombel et al., 2013). This result stems from the ability of VEGF_111_ to freely diffuse from its point of secretion because it is not restricted by extracellular matrix binding. VEGF_111_ has a longer half-life compared to VEGF_165_ and so its actions remain active for longer (Mineur et al., 2007). Thus, a tumor with the ability to secrete several different isoforms, including VEGF_111_, would result in both a highly vascularized tumor and surrounding tissue, potentially increasing the ability to metastasize.

Other VEGF isoforms have been identified in transformed cells including VEGF_145_ (Poltorak et al., 1997) and VEGF_162_ (Lange et al., 2003). VEGF_145_ lacks exons 6b and 7, and is secreted primarily from cancer cell lines derived from the female reproductive system (Poltorak et al., 1997), whereas VEGF_162_ only lacks exon 7 and is secreted by ovarian carcinoma cells (Lange et al., 2003). Both isoforms are angiogenic and will induce angiogenesis *in vitro* and *in vivo* and their biological activity is similar to the other isoforms (Lange et al., 2003). The distinct difference to VEGF_111_ however, is that they still encode the site of plasmin cleavage, and thus lose their biological activity upon exposure to plasmin. Further investigation in mammalian cancers is necessary, however the presence of VEGF_111_ primarily in reproductive tissues of *S. equalis* raises questions whether this isoform may also be present in human reproductive tract cancers.

## Similarities of cancer to invasive placentation

There are similar molecular mechanisms between how tumors invade and metastasize compared to the trophoblastic invasion in hemochorial placentation that occurs in humans, particularly regarding angiogenesis (Murray and Lessey, 1999; Hayakawa, 2006). It has been proposed that there is a positive correlation between the degree of placental invasiveness and the ability of metastatic tumors to develop (D'Souza and Wagner, 2014). Mammals that have evolved to develop the less invasive epitheliochorial and endotheliochorial placentas have a lower observed rate of cancer malignancies. In less invasive forms of placentation, the maternal endometrium limits the invasion of the trophoblast cells, and it is hypothesized that this may correlate with a global suppression of tumor metastasis (Priester and Mantel, 1971; D'Souza and Wagner, 2014).

The discovery of VEGF_111_ as both a potential stimulator of angiogenesis in mammalian tumors (Mineur et al., 2007; Delcombel et al., 2013) and in the uterus of *S. equalis* during pregnancy (Murphy et al., 2010) further supports a connection between placentation and cancer. The fact that *S. equalis* is transitioning between egg laying and giving live birth with a placenta (Stewart et al., 2000) also suggests that this evolutionary process could contribute to an increased susceptibility to cancer. Therefore, an understanding into the role of VEGF_111_ in EC biology may lead to a better understanding of this susceptibility.

## VEGF_111_ as a potential therapeutic agent

The high angiogenic activity of VEGF_111_ could also be used as a potential therapeutic agent to increase vascularization in ischemic diseases and lesions. The presence of plasmin in chronic wounds results in rapid VEGF cleavage (Lauer et al., 2000), but the ability of VEGF_111_ to resist this degradation has recently been exploited to improve ischemia and wound healing (Labied et al., 2013; Kaux et al., 2014). This resistance, as well being freely diffusible, allows VEGF_111_ to induce rapid angiogenesis and rapid EC proliferation compared to the other isoforms, which results in the earlier formation of a functional vasculature and blood supply to the ischemic region (Labied et al., 2013). Further investigation into the clinical applications of VEGF_111_ is necessary, but the initial data are promising.

## Conclusion

VEGF plays a crucial role in normal developmental and embryological angiogenesis as well as contributing to the progression of several pathological conditions. The discovery of VEGF_111_ is an important advance in understanding the mechanisms by which tumors develop their dedicated blood supply and is critical in their ability to grow and metastasize rapidly. While there are several isoforms of VEGF, VEGF_111_ is the only isoform to resist proteolytic degradation and may provide further insights into the pathogenesis and treatment of diseases that are dependent on angiogenesis. Future work on this molecule should be directed at understanding the mechanisms underlying angiogenesis and has great promise in developing new therapeutics to target tumorigenesis and to treat ischemic diseases. This should involve examining the actions of VEGF_111_ on EC biology and its capacity to facilitate the formation of functional vessels in several models to specifically address its role in angiogenesis. This should encompass its actions in cancer, both as a potential contributor to metastasis during disease progression, and drug resistance during disease treatment. Further investigation should also expand on the previous work that focused on VEGF_111_ as a therapeutic agent. Its presence in the uterus of pregnant lizards provides a unique way to understand those mechanisms.

### Conflict of interest statement

The authors declare that the research was conducted in the absence of any commercial or financial relationships that could be construed as a potential conflict of interest.
